# Epigenetic modifications and targeted therapy in pediatric acute myeloid leukemia

**DOI:** 10.3389/fped.2022.975819

**Published:** 2022-09-06

**Authors:** Huan Xu, Yuxi Wen, Runming Jin, Hongbo Chen

**Affiliations:** Department of Pediatrics, Union Hospital, Tongji Medical College, Huazhong University of Science and Technology, Wuhan, China

**Keywords:** acute myeloid leukemia, pediatric, epigenetics, DNA methylation, histone modification, non-coding RNAs, therapy

## Abstract

Acute myeloid leukemia (AML) is a hematological malignancy resulting from the genetic alterations and epigenetic dysregulations of the hematopoietic progenitor cells. One-third of children with AML remain at risk of relapse even though outcomes have improved in recent decades. Epigenetic dysregulations have been identified to play a significant role during myeloid leukemogenesis. In contrast to genetic changes, epigenetic modifications are typically reversible, opening the door to the development of epigenetic targeted therapy. In this review, we provide an overview of the landscape of epigenetic alterations and describe the current progress that has been made in epigenetic targeted therapy, and pay close attention to the potential value of epigenetic abnormalities in the precision and combinational therapy of pediatric AML.

## Introduction

Acute myeloid leukemia (AML) is a blood cancer resulting from the genetic alterations and epigenetic dysregulations of the hematopoietic stem/progenitor cells (1). Pediatric AML accounts for about 15% of children’s leukemias. One-third of children have relapses and approximately half of childhood leukemia-related deaths are caused by relapsed/refractory AML, even though overall survival (OS) has been improved significantly over the past decades (2). Therefore, more specific drugs and more precise therapeutic strategies are urgently needed to improve the outcomes and prolong the survival time of children with AML.

In recent decades, the rapid advancement of sequencing technologies has led to great progress in clarifying the molecular pathogenesis of AML. These advancements lay the ground work and pave the way for precise therapeutic strategy by uncovering genetic alterations and epigenetic dysregulations in AML. Rather than being the result of changes in DNA sequence itself, epigenetic modifications refer to the changes in gene expression inherited through cell division (3). Epigenetic modifications include histone modification, DNA methylation, and non-coding RNAs (ncRNAs), which contribute to initiating and sustaining epigenetic silencing (4). Recent studies have identified a significant role of epigenetic dysregulation in the pathogenesis of AML, and some recurrent somatic genetic alterations in pediatric AML could interfere with epigenetic regulation (5). Epigenetic modifications are frequently reversible, thus offering potential avenues for epigenetic targeted therapy using specific inhibitors (6). Epigenetic therapy has become a promising therapeutic strategy with many novel inhibitors being applied in adult with AML (7). Therefore, it is timely to consider the important role of epigenetic alterations and the potential targeted therapy in pediatric AML, to promote the development of precision therapy and improve the outcomes of children with AML.

## Epigenetic regulation and dysregulation in adult and pediatric acute myeloid leukemia

Multiple epigenetic modifications regulate the transition from hematopoietic stem cells to lineage differentiation and maturation at the transcription level, including DNA methylation, histone modifications, and non-coding RNAs. Epigenetic dysfunction is common in most cancers, and extensive studies have also focused on the mechanisms of epigenetic dysregulation in AML, though AML has few mutations compared to other cancer types (5, 8). The current advances of epigenetic modifications in AML are briefly summarized below (Table 1).

**TABLE 1:** Recurrently mutated or translocated genes in epigenetic modification in adult and pediatric AML.

Gene	Epigenetic function	Type of mutation	Frequency of AML	Prognostic role	References
DNMT3A	*De novo* DNA methylation	Missense, nonsense, and frameshift, 60% heterozygote mutation at R882 residue	20–22% of adult AML; 1–2% of pediatric AML	Adverse prognosis	15–19
TET2	Conversion of 5-methylcytosine to 5-hydroxymethylcytosine	Missense, nonsense, and frameshift mutations	8–23% of adult AML; 1.7% of pediatric AML	Uncertain	16, 22–23
IDH1/IDH2	Conversion isocitrate to α-ketoglutarate (α-KG)	Heterozygous mutations, primarily missense mutations affecting arginine residues	5–33% of adult AML; 1–4% of pediatric AML	Uncertain	16, 27–29
CREBBP	Histone lysine acetyltransferase	Rearrangements (fusion genes)	Rare	Uncertain	14, 33–34
KAT6A	Histone lysine acetyltransferase	Rearrangements (fusion genes)	Rare	Uncertain	14, 33–34
EP300	Histone lysine acetyltransferase	Rearrangements (fusion genes)	Rare	Uncertain	14
HDAC2/HDAC3	Histone deacetylase	Missense mutations	Rare	Uncertain	14
KDM5A	Histone lysine demethylase	Rearrangement involving NUP98	10% of pediatric acute megakaryoblastic leukemia	Uncertain	51, 52
KDM6A	Histone lysine demethylase	Missense mutations	Rare	Uncertain	53
KMT2A	H3K4 methyltransferase	Gene fusion with>70 fusion partners, partial tandem duplications	Fusion: 1–10%; Tandem duplications: 4–7%	Adverse prognosis	39–41
EZH2	H3K27 methyltransferase, enzymatic component of PRC*^a^*2	Missense, nonsense, and frameshift loss-of-function mutations	1–5% of adult AML; Rare in pediatric AML	Uncertain	42, 43
NSD1	H3K36 methyltransferase	Rearrangement involving NUP98	2–5%	Uncertain	36–38
ASXL1	Recruitment of PRC2 to target loci	Missense, nonsense, and frameshift loss-of-function mutations	3–17% of adult AML; 1–9% of pediatric AML	Adverse prognosis, especially in intermediate- and low-risk AML	46, 47
ASXL2	Function unknown	Mutations	23% of AML with RUNX1::RUNX1T1	Uncertain	48, 49
SUZ12	Member of PRC2	Missense mutations, insertions and deletions	Unknown	Uncertain	36
JARID2	Recruit PRC2 to target loci	Loss in transformation of MDS*^b^*/MPN*^c^* to AML	Unknown	Uncertain	45

*^a^*PRC, polycomb repressor complex; *^b^*MDS, myelodysplastic syndrome; *^c^*MPN, myeloproliferative neoplasm.

### DNA methylation

The most well-characterized epigenetic change is DNA methylation, and abnormal methylation patterns have been discovered in gene silencing of tumor suppressor genes and genomic instability (9). The procedure of DNA methylation is the addition of a methyl group to the C5 position of cytosine residues in DNA to form 5-methylcytosine (5-mC). Most CpGs are methylated (70–80%), apart from the CG-dense regions termed CpG islands (CGIs) (10). Malignancy-related aberrant DNA methylation was originally studied in CGIs in gene promoters. Several DNA methyltransferases (DNMTs) and demethylases regulate the methylation modifications of CpGs. The former includes DNMT3A and DNMT3B, while the demethylation procedures are associated with the ten-eleven translocation (TET) family of demethylases (TET1, TET2, and TET3) (11).

Epigenome remodeling is essential for hematopoietic stem cell differentiation and maturation, and there is a direct correlation between DNA methylation patterns and specific cell types during hematopoiesis (12). Epigenetic abnormalities have been identified in AML, and several studies have implicated both hypermethylation and hypomethylation in malignant transformation (13–15). In adult AML, *DNMT3A* is one of the most commonly mutated genes (16), which occurs in pediatric AML at lower frequencies (20–22% vs. 1–2%, respectively) (17). *DNMT3A* mutations were reported to be early events in leukemogenesis and are predominately heterozygous R882H in AML (18). Some studies demonstrated that the *DNMT3A* mutation in hematopoietic stem cells resulted in damaged differentiation, enhanced self-renewal compared to wild-type hematopoietic stem cells, and conferred a poor outcome (19, 20). Meanwhile, *DNMT3A* mutations have been reported to increase chemotherapy resistance and the risk of relapse (21). It is still not well identified how *DNMT3A* mutations result in leukemic transformation, but targeting *DNMT3A* mutations could be a promising therapeutic strategy (22).

*TET2* mutations are another pathway to abnormal DNA methylation. TET2 catalyzes the oxidation procedure of 5-methylcytosine (5-mC) to 5-hydroxymethylcytosine (5-hmC), resulting in DNA demethylation and reversing the gene silencing driven by DNA methylation (23). Mutations in *TET2* occur in 8–23% of patients with AML, but these mutations are observed rarely in children with AML (Table 1) (17). *TET2* mutations are associated with reduced levels of 5-hmC, and are related to a poor prognosis in intermediate-risk AML (24, 25). Li et al. found that *TET2* knockout HSCs were amplified *in vivo* and outperformed wild-type HSCs in serial transplantation assays (26). Besides, Rasmussen et al. demonstrated that *TET2* deletion led to DNA hypermethylation of the active enhancers, which was related to the upregulation of *IGFLR*, *NOTCH3*, and other oncogenes, and the downregulation of tumor suppressor genes, such as *LXN* and *CTDSP1* (27). Subsequently, Shih et al. revealed that *TET2* defect and *FLT3-ITD* mutations remodeled synergistically DNA methylation and gene expression to an extent not seen in either of the mutation alone, including at the GATA2 locus. Then they found that re-expression of GATA2 induced differentiation in AML stem cells and abated leukemogenesis. Consequently, they concluded that the mutations of *TET2* and *FLT3-ITD* induced AML synergistically characterized by site-specific changes in DNA methylation and gene expression (28).

The conversion of isocitrate to α-ketoglutarate (α-KG) is catalyzed by isocitrate dehydrogenase 1/2 (IDH1/2). The conversion of 5-mC to 5-hmC and subsequent DNA demethylation is also the result of combined effect of IDH1/2 and TET2 (29). Mutations in *IDH1/2* are frequently observed in adult AML (5–33%), less observed in pediatric AML (1–4%) (17, 30, 31). Mutations in *IDH1/2* result in the synthesis of tumor metabolite 2-hydroxyglutarate (2-HG), leading to aberrant DNA methylation (32). Mutational epigenetic profiling revealed that AML cells with *IDH1/2* mutations showed global DNA hypermethylation and a specific hypermethylation characteristic, particularly at promoter regions in a large AML patient cohort study (33). Figueroa et al. found that *IDH1/2* mutations and *TET2* mutations were mutually repellent, and loss-of-function mutation of *TET2* was similar to the epigenetic alterations of *IDH1/2* mutations. Furthermore, either *TET2* depletions or *IDH1/2* mutations increased progenitor cell marker expression and damaged hematopoietic differentiation, cooperatively contributing to leukemogenesis (33). Similar to AML with *TET2* or *IDH1/2* mutations, Rampal et al. identified reduced 5-hmC levels in *WT1* mutant AML patients and they found that the overexpression of WT1 increased global levels of 5-hmC, whereas 5-hmC levels were reduced when *WT1* was silenced (34). They also demonstrated that WT1 physically interacts with TET2 and TET3, and loss-of-function WT1 caused a hematopoietic differentiation phenotype similar to that observed with *TET2* defects (34). Subsequently, Wang et al. also demonstrated that WT1 physically interacts with and recruits TET2 to its target genes, and AML-derived *TET2* mutations disrupt the interaction (35). Despite the mechanism by which *WT1* silencing or mutations lead to decreased 5-hmC is not completely clarified, the *TET2*, *IDH1/2*, and *WT1* mutations lead to dysregulated DNA hydroxymethylation, which could be classified as a new subtype of AML.

### Histone acetylation

Histone acetylation is the transformation of acetyl groups to lysine residues in histone proteins, which is modulated by histone lysine acetyltransferases (KATs) and histone deacetylases (HDACs). Histone acetylation plays an important role in gene transcription, chromatin structure, and DNA repair (36). Lysine acetylation leads to open chromatin confirmations and gene activation, whereas lysine deacetylation leads to condensed and closed chromatin, causing gene inactivation (37). KAT rearrangements (as opposed to mutations) occur in AML at extremely low frequencies (15). Although HDACs mutations occur also exceptionally rarely in children with AML, myeloid oncoproteins and leukemia-associated fusions can recruit HDACs abnormally, such as *EVI1*, RUNX1::RUNX1T1 (previously AML1::ETO), so as to block differentiation and maintain the leukemic phenotype of AML (38).

### Histone lysine methylation and demethylation

Histone lysine methylation is regulated by lysine methyltransferases (KMTs), which have several different degrees of methylation, including monomethylation, dimethylation, and trimethylation (39). It has been reported that the methylation of histone lysine could change the affinity of reader proteins to the methylated histone (40). The different target residues and the degree of methylation have distinct effects on transcription levels, and different methylation states could produce different functional effects in the same lysine residue (41). For instance, activation-related marks include methylation of H3K4, H3K79, and H3H36, while the methylation of H3K27, H3K9, and H4K20 contributes to silenced gene transcription (41). In AML, KMTs include components of the polycomb repressor complexes (PRCs) and mixed-lineage leukemia (MLL) proteins, which are frequently involved in translocations or are mutated.

MLL, or called KMT2A belongs to the family of SET domain-containing KMTs. MLL can make transcription activation by targeting H3K4. In AML, a histone methyltransferase DOT1 is recruited by MLL fusion proteins, so as to cause abnormal methylation of H3K79 at MLL gene targets and increase the expression of leukemia-related genes (42). MLL translocations are more common in pediatric than adult AML (30–50% vs. >10%, respectively), which are the most frequent alteration in infant AML (43, 44). Therefore, it is a promising area to develop the inhibitors of this complex and its enzymatic co-factors for infant and pediatric AML.

Enhancer of zeste 2 polycomb repressive complex 2 subunit (EZH2) is part of the PRC2 polycomb repressor complex, which is an H3K27 methyltransferase to catalyze dimethylation and trimethylation of H3K27, resulting in the suppression of transcription (45, 46). Neff et al. found that EZH2 was necessary for tumor progression rather than leukemogenesis in KMT2A::MLLT3 (previously MLL::AF9) leukemia (47). Another member of the PRC2 complex Jumonji AT-rich interactive domain 2 (JARID2) similarly recruits PRC2 to specific target loci. Puda et al. reported that deletions of PRC2 complex members, especially *JARID2*, contribute to the leukemic transformation of chronic myeloid disorders (48). Additional sex combs like transcriptional regulator 1/2 (*ASXL1/2*) mutations occur more frequently in adult than pediatric AML (3–15% vs. 1–9%, respectively) (49, 50). The PRC2-associated protein ASXL1 can recruit PRC2 to its target loci, and mutations of *ASXL1* lead to deletion of methylation regulated by PRC2. It has been reported that *ASXL1* mutations contributed to the poor prognosis of AML (51). Besides, mutations of *ASXL2* are commonly found in patients with the *RUNX1::RUNX1T1* fusion gene, and *ASXL1* and *ASXL2* mutations are mutually exclusive (52).

Histone lysine demethylation is regulated by lysine demethylases (KDMs), which remove the methylation labels on lysine. KDMs include the amine oxidases and Jumonji domain containing proteins (JmjC). Lysine-specific demethylase 1 (LSD1/KDM1A) is the amine oxidase, which has specificity for H3K4 and H3K9 as a transcriptional activator or a transcriptional repressor (53). The demethylase of JmjC lysine, KDM5A (JARID1), is associated with NUP98 fusion protein in about 10% of pediatric acute megakaryoblastic leukemia (54, 55), and the mutation of *KDM6A*, another member of the JmjC family, occurs rarely in AML (56).

### Epigenetic readers

As epigenetic readers, the bromodomain and extra-terminal (BET) protein family members, including BRD2, BRD3, and BRD4, bind to acetylated lysine residues on histone tails (57). The process can initiate chromatin-mediated signal transduction, so as to achieve normal or tumor-dependent functions (58, 59). Some studies have reported that BRD4 could promote the abnormal expression of pivotal oncogenes, including *c-Myc* and *Bcl-2* in AML (60, 61). BET inhibitors can alter the expression of particular genes, despite BRD4 and other BET proteins ubiquitously express at gene promoters and enhancers (62). Zuber et al. indicated that inhibition of BET proteins can block abnormal transcription of some oncogenes associated with leukemia, thus blocking the upregulation of leukemic stem cell (LSC) self-renewal programs and inducing differentiation (63). Furthermore, Dawson et al. demonstrated that BET inhibition could exhibit profound anti-leukemic effects against human and murine MLL-fusion leukemic cell lines, and they also identified the effects in mouse models of murine KMT2A::MLLT3 and human KMT2A::AFF1 (previously MLL::AF4) leukemia (64). Inhibition of tumor cell dependence on high oncogene expression by BET inhibitors is a promising therapeutic strategy, and malignant cells may be eliminated in the therapeutic window that preserves normal hematopoietic cells.

### Non-coding RNAs

With the rapid advancement of RNA sequencing technology, more and more ncRNA have been discovered that are closely associated with AML leukemogenesis. There is accumulating evidence that ncRNAs play an important role in the pathogenesis of hematological malignancies, especially AML (65). Over the last few decades, the understanding of ncRNAs promote the improvement of the diagnosis, treatment, and prognosis of AML. NcRNA can be classified as housekeeping RNA and regulatory RNA according to their different functions. The regulatory RNA molecules are widely associated with gene transcription and translation, which include microRNAs (miRNAs, 19–24 bp), long non-coding RNAs (lncRNAs, >200 bp), and circular RNAs (circRNAs) (66).

MiRNAs have about 22 nucleotides that bind to the 3’-untranslated regions (3’-UTR) of the target gene and posttranscriptionally suppress the expression level of the target gene (67). Numerous studies have implicated miRNAs in regulating hematopoiesis. Oshima et al. demonstrated that EZH2 cooperated with miRNA let-7 to inhibit HSC function (68). Bolouri et al. performed miRNA sequencing of 152 samples from pediatric AML patients and found a relationship between gene abnormalities and miRNA expression. They found the high expression of miRNA-10a in AML with the mutations of NPM1 and high miRNA-21 expression in Core Binding Factor (CBF)-AMLs (69). Some studies have shown that miRNA-155 was associated with poor prognosis of adult and pediatric AML (70). Zhu et al. analyzed the connection of the miRNA data and clinical data of 229 patients and verified that the high expression of has-miR-542 and has-miR-509 were independent adverse prognostic factors, while has-miR-146a and has-miR-3667 were favorable factors (71).

CircRNAs are ubiquitous, stable, and conserved ncRNAs, which are single-stranded RNA molecules (72). Nicolet et al. demonstrated that the expression of circRNA is cell-type specific and increases during hematopoietic differentiation after analyzing circRNA expression in human hematopoietic progenitors and differentiated lymphoid and myeloid cells (73). Liu et al. utilized a circRNA microarray to analyze the expression pattern of circRNAs in children with AML. Then they verified that circRNF220 was specifically enriched and accumulated in peripheral blood and bone marrow of children with AML (74). Subsequently, they showed that circRNF220 has highly specificity and efficiency in the diagnosis of AML. Meanwhile, they demonstrated that the expression of circRNF220 independently predicted prognosis and high expression of circRNF220 was an adverse prognostic marker for relapse of children with AML. Recently, Wang et al. showed that circ_0040823 sponged miR-516b to inhibit proliferation and induced apoptosis of AML cells (75).

LncRNAs are more than 200 nucleotides in length and lack a meaningful open reading frame (76). Relatively fewer studies have investigated lncRNAs in pediatric AML. It has been reported that urothelial carcinoma-associated 1(UCA1) could maintain the proliferation of AML cells (77, 78). Recently, Liang et al. found that the expression of UCA1 was increased and the expression of miR-204 was inhibited in pediatric patients with AML. Besides, the downregulation of UCA1 suppressed cell proliferation and facilitated apoptosis by upregulating miR-204 in pediatric AML (79). Ma et al. identified that lncRNA LINC00909 promoted cell proliferation and metastasis by miR-625-mediated modulation of the Wnt/β-catenin signal pathway in pediatric AML (80).

The role of miRNAs in the pathogenesis and prognosis of AML is the most studied. However, the mechanism of miRNAs in AML is still complicated and poorly understood. Recently, lncRNAs and circRNAs have been found to participate in the miRNA network and act as competing endogenous RNAs (ceRNAs) and miRNA sponges to regulate the expression of miRNAs in AML (65). It is a promising area to find the crossover of the three ncRNAs to help understand the connection between these three ncRNAs.

## Epigenetic targeted therapy in pediatric acute myeloid leukemia

The pivotal role of epigenetic modifications in AML has stimulated efforts to study epigenetically targeted drugs. Furthermore, epigenetic targeted therapies provide more chances for patients with AML thanks to the inherent reversibility of epigenetic marks. Numerous clinical trials are ongoing to study the epigenetic targeted therapies in adults with AML, either alone or combinational therapy. Despite mutations of epigenetic modifications are observed less commonly in pediatric AML compared to adult AML, more and more clinical trials are focusing on the important role of epigenetic modifications and targeted therapy in pediatric AML, and a series of small molecules that inhibit epigenetic regulators activity are currently studied in various clinical trial stages (Table 2).

**TABLE 2:** Clinical trials of epigenetic targeted therapies in pediatric AML.

Target	Drug	Phase	Study start	Clinical trial	Status
DNA methyltransferases	Azacitidine	1	2013	NCT01861002	Completed
	Azacitidine	2	2017	NCT03164057	Recruiting
	Azacitidine	2	2018	NCT03383575	Active, not recruiting
	Azacitidine	2	2015	NCT02450877	Completed
	Azacitidine	2	2013	NCT01700673	Completed
	Azacitidine	2	2014	NCT02275663	Unknown
	Decitabine	2	2017	NCT03164057	Recruiting
	Decitabine	1	2017	NCT03132454	Recruiting
	Decitabine	2	2006	NCT00416598	Completed
	Decitabine	1/2	2018	NCT03453255	Unknown
	Decitabine	2	2018	NCT03417427	Recruiting
	Decitabine	2	2006	NCT00414310	Completed
	Decitabine	1	2017	NCT03263936	Active, not recruiting
	Decitabine	1/2	2013	NCT01853228	Terminated
	Decitabine	2	2011	NCT01177540	Completed
IDH1	Ivosidenib		2017	NCT03245424	Approved for marketing
IDH2	Enasidenib	2	2018	NCT03383575	Active, not recruiting
Histone deacetylases	Vorinostat	1	2005	NCT00217412	Completed
	Vorinostat	1	2017	NCT03263936	Active, not recruiting
	Vorinostat	1/2	2012	NCT01422499	Completed
	Panobinostat	1	2016	NCT02676323	Terminated
	Panobinostat	1	2011	NCT01321346	Completed
	Valproic acid	2	2012	NCT02124174	Recruiting
DOT1L	Pinometostat	1	2014	NCT02141828	Completed
	Pinometostat	1/2	2019	NCT03724084	Active, not recruiting

### DNA methyltransferase inhibitors

**FIGURE 1:**
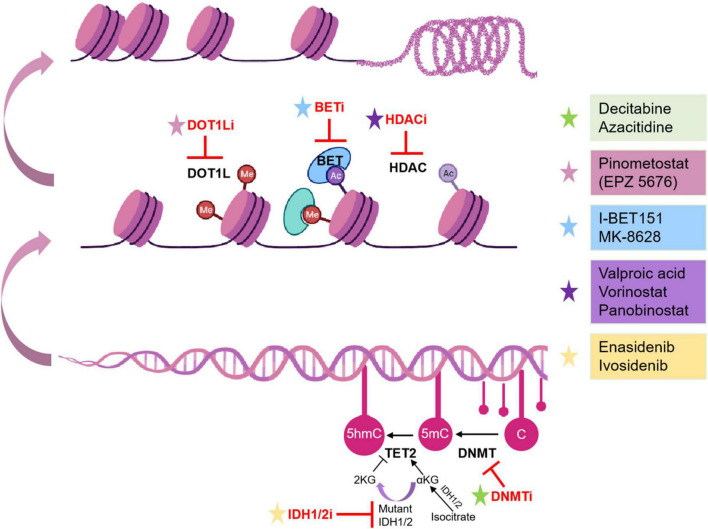
Epigenetic inhibitors and mechanisms in pediatric AML. Epigenetic inhibitors are highlighted in red. C, cytosine; Ac, acetylation.

DNA methyltransferase inhibitors (DNMTi) or called hypomethylating agents (HMAs) include 5-azacytidine (azacitidine) and 5-aza-2’-deoxycytidine (decitabine). HMAs can alter DNA methylation patterns, promote the expression of tumor suppressors, and increase apoptosis (81). HMAs are the best-established epigenetic therapies in adult AML, which have shown efficacy and safety in older patients with AML. HMAs covalently bond with DNMTs irreversibly, which leads to proteasomal degradation of DNMTs, resulting in hypomethylation and transcriptional repression, and direct cytotoxic effects through DNA damage (Figure 1) (82). Several studies have demonstrated that the action of azacitidine is not limited to DNA demethylation (83). Schaefer et al. found that azacitidine inhibited the RNA methyltransferase DNMT2, which is variably expressed in human cancer cell lines (84). Subsequently, some studies have identified the novel targets of azacitidine in RNA methylation, which provides a new insight into its more widespread clinical use either or in combination in AML (85, 86). HMAs have been demonstrated to prolong overall survival (OS) compared to standard treatment in adult patients (87), and Stahl et al. identified the important role of HMAs in relapsed/refractory AML in a large patient cohort study (88). Despite the low frequency of mutations in DNMTs in pediatric AML, some studies have shown that DNMTi might be efficient in pediatric AML. Gore et al. demonstrated that decitabine is feasible and well- tolerated in children with newly diagnosed AML before standard combination chemotherapy (89). Subsequently, Sun et al. identified that azacitidine was safe when utilized in sequence with intensive chemotherapy in pediatric relapsed/refractory AML (90). Some studies have indicated that HMAs only have limited efficacy and difficulty leading to sustained remission when used as a single agent in adult AML (91), thus further preclinical and clinical studies should focus on the combination therapy with other chemotherapy agents, targeted drugs, and immunotherapy.

### Isocitrate dehydrogenase inhibitors

IDH mutations have been identified to block cell differentiation by competitively inhibiting α-KG-dependent dioxygenases involved in histone and DNA demethylation (92, 93). Current IDH inhibitors selectively inhibit mutant IDH protein and promote the terminal differentiation of abnormal myeloid cells *in vitro* and *in vivo* models (94). Stein et al. firstly reported the IDH inhibitor, enasidenib, could be used in relapsed/refractory *IDH2*-mutant AML in a phase 1/2 clinical trial (NCT01915498) (95). Then DiNardo et al. reported that ivosidenib, a small-molecule inhibitor of IDH1, promoted durable remission and molecular remission in patients with CR in a phase 1 clinical trial of patients with advanced *IDH1*-mutant relapsed/refractory AML (96). However, few clinical trials accept children with AML at present. The only clinical trial currently that accepts pediatric AML patients is investigating the synergistic effect of azacytidine and enasidenib in myelodysplastic syndrome (MDS) with *IDH2* mutation (NCT03383575), whereas there is no ongoing trial in pediatric AML yet.

### Histone deacetylases inhibitors

HDAC inhibitors (HDACi) can activate tumor suppressor genes and promote tumor cell killing, which have been evaluated in clinical trials for adult AML patients with limited efficacy, either alone or in combination with chemotherapy (97). Leukemia-associated fusion proteins have been reported to block gene expression by recruitment of HDACs, which could be alleviated by inhibiting HDACs, causing differentiation of leukemic blasts. Meanwhile, these fusion proteins are more frequent in pediatric AML, raising the possibility that children with AML may benefit more from HDAC inhibitors than adult patients. Karol et al. reported that panobinostat could be safely administrated with chemotherapy and increased histone acetylation in a phase 1 clinical trial (98). Recently, Pommert et al. demonstrated that the combination therapy of decitabine and vorinostat with fludarabine, cytarabine, and G-CSF (FLAG) was well-tolerated and effective in pediatric patients with relapsed/refractory AML in phase 1 clinical trial (NCT02412475) (99). The results of these clinical trials will promote the development of HDAC inhibitors in pediatric patients with AML.

### DOT1L inhibitors

Owing to more frequent MLL translocations of pediatric AML than adult AML (30–50% vs. >10%, respectively), highly promising therapeutic approaches can be applied in MLL-associated leukemias. Inhibition of DOT1L blocks MLL target gene expression by regulating the aberrant methylation of H3K79. Pinometostat (EPZ-5676) is a DOT1L inhibitor that has been applied in relapsed/refractory AML patients with MLL rearrangements in a phase 1 clinical trial (100). However, the study reported only temporary reductions in leukemic blasts (NCT02141828). Despite the lack of clinical benefit of EPZ-5676 as a single agent, preclinical and clinical studies are warranted to evaluate the combinational efficacy of DOT1L inhibitors and conventional regimens in relapsed/refractory AML.

### Bromodomain and extra-terminal inhibitors

BET inhibitors have an important role in inhibiting histone acetylation. Dawson et al. showed that I-BET151, a small-molecule inhibitor of the BET family, was effective against murine and human leukemic cell lines with MLL-fusion by inducing early cell cycle arrest and apoptosis (64). Then they indicated that IBET151 significantly promoted survival in two distinct mouse models of murine KMT2A:MLLT3 and human KMT2A::AFF1leukemia (64). These results provide a promising epigenetic therapy target for pediatric AML. Other BET inhibitors, including MK-8628 (OTX015) and CPI-0610, also are now being evaluated in phase 1 and 2 trials in adult AML (NCT02698189, NCT02158858) (101).

## Discussion

Although the prognosis for children with AML has improved in recent decades, it remains poor due to the high risk of relapse and few therapeutic choices available when initial treatment fails. There remains an urgent need for better and more precise therapeutic approaches for patients with AML, especially for pediatric AML. Rapid advancements of next-generation sequencing technologies have contributed to understanding genetic alterations and epigenetic abnormalities of AML, and promoting the development of precise therapeutic strategies. Based on the clinical efficacy of epigenetic therapies in adult AML, the preclinical and clinical study of epigenetic targeted therapy needs more attention, despite the low frequency of epigenetic alterations in children with AML. Some inhibitors of epigenetic targeted therapies have benefited many children with the appropriate patient stratification. Meanwhile, it is critical that more personalized medicine will need more precise and appropriate patient stratification with different genetic and epigenetic alterations. One drug is unlikely to be curative in AML, either epigenetic targeted drugs or other inhibitors, which give opportunities for drug resistance and increase the risk of relapse due to the clonal heterogeneity. Several clinical trials have demonstrated that the Bcl-2 inhibitor, venetoclax plus HMAs dramatically improved CR rates in elderly AML patients (102). And venetoclax plus azacitidine was approved by FDA in newly diagnosed AML ineligible for induction chemotherapy in 2018. Recently, IDH1 inhibitor, ivosidenib in combination with azacitidine was also approved for newly diagnosed AML (103). Further preclinical and clinical studies should focus on the combination therapeutic strategies of epigenetic targeted drugs with other inhibitors and immunotherapy to promote cell killing and improve the prognosis for children with AML.

## Author contributions

HX and YW collected the data and wrote the manuscript. HC and RJ reviewed and finalized the manuscript. All authors have read and agreed to the published version of the manuscript.
